# Supplementary material for: The yeast mitochondrial pyruvate carrier is a hetero‐dimer in its functional state
Source: EMBO J. 2019 Apr 12;38(10):e100785. doi: 10.15252/embj.2018100785 (PMC6517818; doi:10.15252/embj.2018100785)
Supplement: Supplementary file 1 — Expanded View Figures PDF [file EMBJ-38-e100785-s001.pdf]

Expanded View Figures

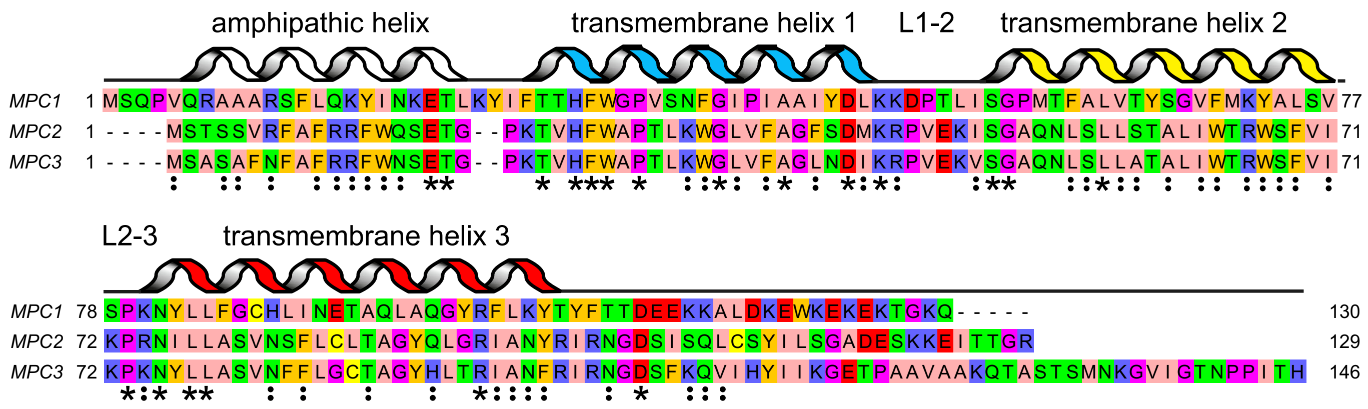

Figure EV1. Topology of yeast Mpc1, Mpc2 and Mpc3.

The alignment was generated by Clustal Omega and by manual curation (Sievers *et al*, 2011). The aligned residues are coloured by the ZAPPO colour scheme in which aliphatic, polar, aromatic, positively charged, negatively charged, Pro/Gly and Cys are coloured pink, green, orange, blue, red, magenta and yellow, respectively. The asterisks indicate identical residues and the colon conserved substitutions. Also indicated are putative transmembrane helices, loop regions and the N-terminal amphipathic helix. The secondary structure elements were assigned based on PSIPRED (Buchan *et al*, 2013), MEMSAT3 (Jones *et al*, 1994) and conservation analysis.

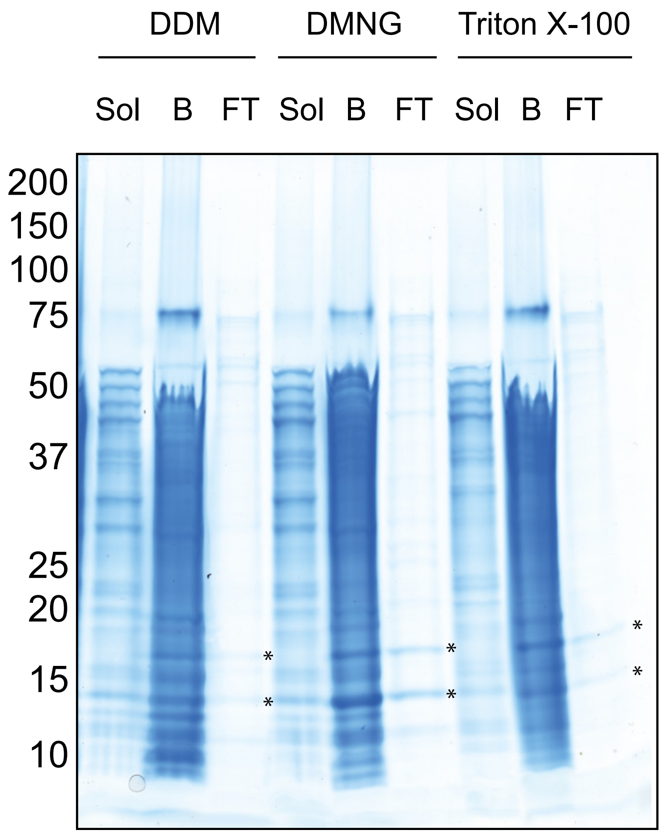

Figure EV2. The Mpc1/Mpc3 hetero-complex purified in different detergents.

The Mpc1/Mpc3p hetero-complex was purified in *n*-dodecyl β-D-maltoside (DDM), decyl maltose neopentyl glycol (DMNG) or Triton X-100. The solubilisation of mitochondria was performed as under Materials and Methods but in buffer containing 2% (w/v) DDM or DMNG or 1% (w/v) Triton X-100. For affinity purification, the nickel Sepharose columns were washed with wash buffers containing 0.1% (w/v) DDM, DMNG or 0.1% (w/v) Triton X-100 supplemented with 0.1 mg/ml tetraoleoyl cardiolipin (TOCL). The samples are the solubilisate of mitochondria (Sol), proteins remaining on the resin after Factor Xa cleavage (B) and flow-through from the resin (FT). Asterisks indicate the Mpc1 and Mpc3 proteins.

© 2019 The Authors

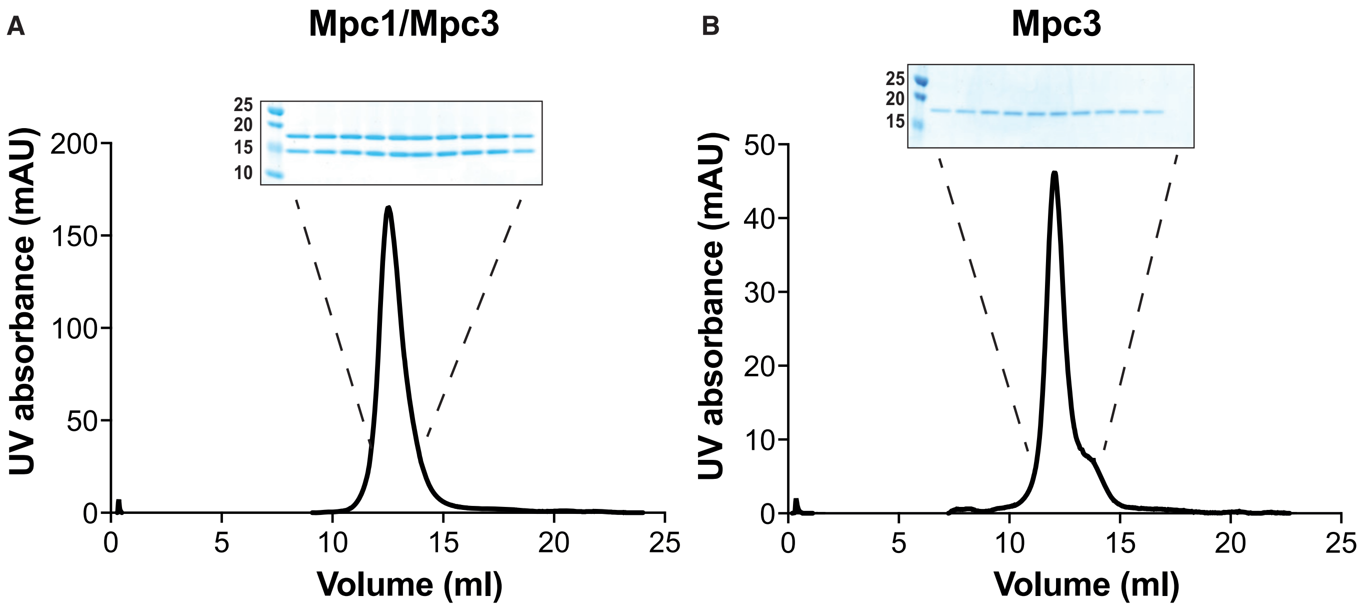

Figure EV3. Size-exclusion chromatography.

A Size-exclusion chromatography of 500 μg nickel-affinity-purified Mpc1/Mpc3 hetero-complex. In the A_{280} profiles, the Mpc1/Mpc3 hetero-complex was present in a symmetrical peak.

B Size-exclusion chromatography of 150 μg nickel-affinity-purified Mpc3. Mpc3 produced a peak similar to that of Mpc1/Mpc3.

Data information: Insets in (A and B) show peak fractions collected and analysed by SDS–PAGE and visualised by Coomassie Blue stain.

© 2019 The Authors

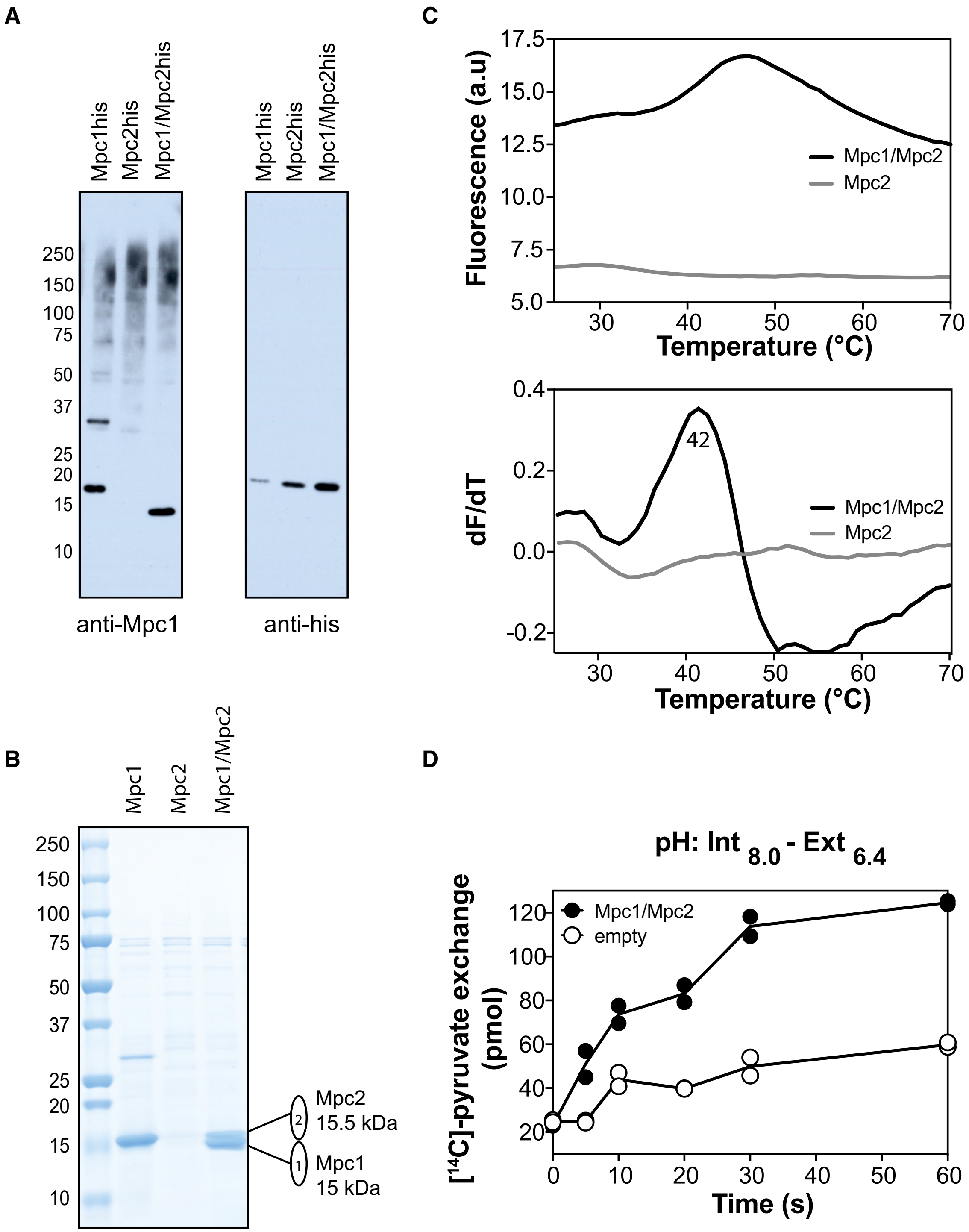

Figure EV4. Purification and stability analysis of the hetero-complex Mpc1/Mpc2.

A The individual histidine-tagged Mpc1 (Mpc1his), histidine-tagged Mpc2 (Mpc2his) or the Mpc1/Mpc2 hetero-complex (Mpc1/Mpc2his) was expressed in the triple *mpc* knock-out strain SHY15 and detected with antibodies raised against Mpc1 (left) or against the histidine tag (right).

B SDS–PAGE analysis of purified Mpc1, Mpc2 and Mpc1/Mpc2 proteins, visualised with Coomassie Blue stain. As the yield of the purified Mpc2 was very low, the protein was not visible with Coomassie Blue stain, but was identified on the gel by peptide mass finger printing (Table EV1).

C The stability of the purified Mpc1/Mpc2 or Mpc2 was assessed via the CPM method. Thermal denaturation profiles (*upper panel*) were used to calculate the first derivative of the data (*lower panel*). The number is the apparent melting temperature for the Mpc1/Mpc2 hetero-complex.

D Time course of pyruvate homo-exchange by the Mpc1/Mpc2 hetero-complex in liposomes in comparison with empty liposomes at a ΔpH of 1.6 (n = 2).

© 2019 The Authors

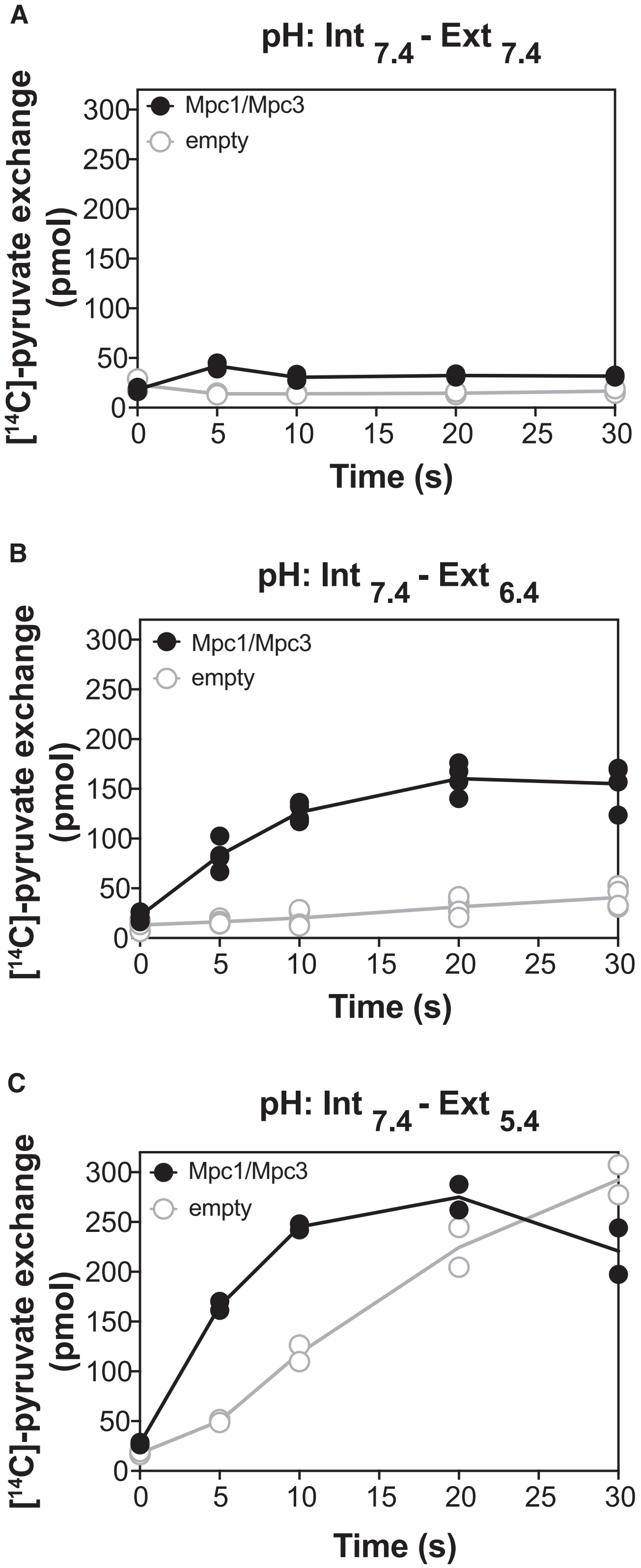

Figure EV5. Effect of pH on pyruvate homo-exchange in Mpc1/Mpc3-containing proteoliposomes compared to diffusion of pyruvate into empty liposomes.

Pyruvate homo-exchange of Mpc1/Mpc3 proteoliposomes or empty liposomes was tested in three conditions.

A In the absence of a ΔpH, using an internal buffer pH of 7.4 and external buffer pH of 7.4 (n = 2).

B At a ΔpH of 1.0, using an internal buffer pH of 7.4 and external buffer pH of 6.4 (n = 4).

C At a ΔpH of 2.0, using an internal buffer pH of 7.4 and external buffer pH of 5.4 (n = 2).

© 2019 The Authors
