# Supplementary material for: The yeast mitochondrial pyruvate carrier is a hetero‐dimer in its functional state
Source: EMBO J. 2019 Apr 12;38(10):e100785. doi: 10.15252/embj.2018100785 (PMC6517818; doi:10.15252/embj.2018100785)
Supplement: Supplementary file 2 — Table EV1 [file EMBJ-38-e100785-s002.docx]

**Table EV1 - Peptide mass fingerprinting.**

| **Protein** | **Peptides Identified** | | |
| --- | --- | --- | --- |
| **Mpc1** | **Mpc1**  YALSVSPK (35, -18ppm)  YTYFTTDEEK (49, 3ppm)  YTYFTTDEEKK (83, 19ppm)  NYLLFGC(Pam)HLINETAQLAQGYR (100,31ppm)  YIFTTHFWGPVSNFGIPIAAIYDLK (79, -16ppm) | | **Mpc1 dimer**  YTYFTTDEEK (38, 1ppm)  YTYFTTDEEKK (78, 13ppm) |
| **Mpc2** | TVHFWAPTLK (62, -9ppm)  WGLVFAGFSDM (Ox) K (35, -14ppm)  ISGAQNLSLLSTALIWTR (50, 7ppm)  WSFVIKPR (29,32ppm) | | |
| **Mpc3** | SASAFNFAFR (71, 37ppm)  FWNSETGPK (63, 10ppm)  TVHFWAPTLK (72, 16ppm)  WGLVFAGLNDIK (116, 27ppm)  VSGAQNLSLLATALIWTR (151, 73ppm)  WSFVIKPK (46, -2.65ppm)  NYLLASVNFFLGC(Pam) TAGYHLTR (102, 45ppm) | | |
| **Mpc1/Mpc2** | **Mpc1**  YALSVSPK (31,22ppm)  YTYFTTDEEK (59, -9ppm)  YTYFTTDEEKK (85, 4ppm)  NYLLFGC(Pam)HLINETAQLAQGYR (133, 34ppm) | **Mpc2**  RFWQSETGPK (58,64ppm)  FWQSETGPK (67,9ppm)  TVHFWAPTLK (58, 30ppm)  WGLVFAGFSCMK (38, 31ppm)  WGLVFAGFSCM (Ox) K (88, 32ppm)  ISGAQNLSLLSTALIWTR  WSFVIKPR (157, 70ppm) | |
| **Mpc1/Mpc3** | **Mpc1**  YALSVSPK (33, 9ppm)  NYLLFGC(Pam)HLINETAQLAQGYR (115, 48ppm)  YTYFTTDEEK (46, -6ppm)  YTYFTTDEEKK (83, 9ppm) | **Mpc3**  SASAFNFAFR (74, 68ppm)  FWNSETGPK (62, 11ppm)  TVHFWAPTLK (72, 39ppm)  WGLVFAGLNDIK (125, 66ppm)  VSGAQNLSLLATALIWTR (146, 113ppm)  WSFVIKPK (41, -30ppm) | |

^Numbers in parentheses are, respectively, peptide ion score and experimentally observed difference of ion mass from expected m/z. C(Pam); propionamide cysteine (acrylamide adduct), M(Ox); methionine oxide.^
