# Supplementary material for: The yeast mitochondrial pyruvate carrier is a hetero‐dimer in its functional state
Source: EMBO J. 2019 Apr 12;38(10):e100785. doi: 10.15252/embj.2018100785 (PMC6517818; doi:10.15252/embj.2018100785)
Supplement: Supplementary file 3 — Table EV2 [file EMBJ-38-e100785-s003.docx]

**Table EV2 - Summary of SEC-MALLS data.**

|  | Experimentally determined molecular weights (kDa) | | |
| --- | --- | --- | --- |
| Purification method | Protein-detergent-lipid complex (PDL) | Detergent-lipid (DL) | Protein (P) |
| **Mpc1/Mpc3** | | | |
| Nickel affinity | 169.6 ± 1.9 | 139.5 ± 2.4 | 30.14 ± 0.3 |
| Nickel affinity | 163.9 ± 1.8 | 130.7 ± 2.4 | 33.21 ± 0.4 |
|  |  |  |  |
| Nickel affinity/SEC | 155.6 ± 0.9 | 124.9 ± 1.3 | 30.69 ± 0.2 |
| Nickel affinity/SEC | 159.8 ± 2.2 | 129.8 ± 2.9 | 30.04 ± 0.4 |
| Nickel affinity/SEC | 167.8 ± 1.2 | 137.7 ± 1.5 | 30.08 ± 0.2 |
| **Mpc3** | | | |
| Nickel affinity | 179.8 ± 3.2 | 145.9 ± 4.2 | 33.99 ± 0.6 |
| Nickel affinity | 191.9 ± 2.3 | 156.8 ± 3 | 35.17 ± 0.4 |
| Nickel affinity | 176.9 ± 2.7 | 141.6 ± 3.4 | 35.32 ± 0.5 |
